# Glucocorticoid treatment in early rheumatoid arthritis is independently associated with increased PCSK9 levels: data from a randomised controlled trial

**DOI:** 10.1136/rmdopen-2024-005129

**Published:** 2025-06-05

**Authors:** Kristina Lend, Jos WR Twisk, Nupur Kumar, Bas Dijkshoorn, Jon Lampa, Anna Rudin, Merete Lund Hetland, Till Uhlig, Dan Nordström, Mikkel Østergaard, Bjorn Gudbjornsson, Tuulikki Sokka-Isler, Gerdur Grondal, Kim Hørslev-Petersen, Michael T Nurmohamed, Johan Frostegård, Ronald F van Vollenhoven

**Affiliations:** 1 Department of Rheumatology and Clinical Immunology, Amsterdam University Medical Centers, Amsterdam, The Netherlands; 2 Division of Rheumatology, Department of Medicine, Karolinska Institute, Stockholm, Sweden; 3 Department of Epidemiology and Data Science, Amsterdam University Medical Centers, Amsterdam, The Netherlands; 4 Unit of Immunology and Chronic Disease, Institute of Environmental Medicine, Karolinska Institute, Stockholm, Sweden; 5 Amsterdam Rheumatology and immunology Center, Reade, Amsterdam, The Netherlands; 6 Department of Gastroenterology, Dermatology and Rheumatology, Karolinska University Hospital, Stockholm, Sweden; 7 Department of Rheumatology and Inflammation Research, Institute of Medicine, The Sahlgrenska Academy, University of Gothenburg, Sahlgrenska University Hospital, Gothenburg, Sweden; 8 Center for Rheumatology and Spine Diseases, Rigshospitalet Glostrup, Glostrup, Denmark; 9 University of Copenhagen, Faculty of Health and Medical Sciences, Copenhagen, Denmark; 10 Center for Treatment of Rheumatic and Musculoskeletal Diseases (REMEDY), Diakonhjemmet Hospital, Oslo, Norway; 11 University of Oslo, Oslo, Norway; 12 Department of Medicine, Helsinki University and Helsinki University Hospital, Helsinki, Finland; 13 Center for Rheumatology and Spine Diseases, Glostrup, Denmark; 14 Centre for Rheumatology, Centre for Rheumatology Research, University Hospital and Faculty of Medicine, University of Iceland, Reykjavik, Iceland; 15 University of Eastern Finland, Kuopio, Finland; 16 Wellbeing services county of Central Finland, Jyväskylä, Finland; 17 Landspitali University Hospital, Reykjavk, Iceland; 18 University Hospital of Southern Denmark, Sønderborg, Denmark; 19 Department of Regional Health Research, University of Southern Denmark, Odense, Denmark; 20 Institute of Environmental Medicine, Karolinska Institute, Stockholm, Sweden; 21 Department of Rheumatology, Amsterdam Rheumatology and Immunology Center, Amsterdam, The Netherlands

**Keywords:** Glucocorticoids, Lipids, Cardiovascular Diseases, Arthritis, Rheumatoid, Antirheumatic Agents

## Abstract

**Background:**

Rheumatoid arthritis elevates cardiovascular disease risk. Proprotein convertase subtilisin/kexin type 9 (PCSK9), a regulator of low-density lipoprotein (LDL) metabolism, increases LDL-receptor breakdown in the liver, which elevates LDL-cholesterol levels. In addition, PCSK9 has direct effects on thrombogenesis and atherosclerotic plaque formation.

We aimed to investigate (1) the impact of glucocorticoids and biological disease-modifying antirheumatic drug (bDMARD) treatments on PCSK9 and LDL-cholesterol levels, (2) whether this influence is different when autoantibodies are present and (3) the association between PCSK9 and LDL cholesterol.

**Methods:**

In this post hoc analysis of the NORD-STAR trial, 296 newly diagnosed patients starting methotrexate with glucocorticoids, certolizumab pegol, abatacept or tocilizumab were included. Serum PCSK9 and LDL-cholesterol levels were measured at baseline and 24 weeks. Linear regression models were used to analyse the difference in PCSK9 and LDL cholesterol between glucocorticoid and bDMARD treatments at 24 weeks. In the second analysis, the interactions between the treatment groups and autoantibody status were added to the model.

**Results:**

After 24 weeks, PCSK9 levels were higher in the glucocorticoid group than in the combined bDMARD treatment group (−276.0 (95% CI −468.2 to −83.9)). When compared with the bDMARD treatment, these increases were more pronounced in autoantibody-positive patients. Changes in LDL cholesterol exhibited a pattern distinct from PCSK9, as it increased in all treatments.

**Conclusion:**

Glucocorticoid treatment was associated with increased PCSK9 levels after 24 weeks. When compared with the bDMARD treatments, these increases were more pronounced in rheumatoid factor, anticitrullinated protein antibody and antinuclear antibody-positive patients. Our data provide a potential mechanistic link between glucocorticoid treatment and cardiovascular disease.

**Funding:**

Inger Bendix Foundation for Medical Research.

**Trial registration number:**

EudraCT2011-004720-35, NCT01491815.

**WHAT IS ALREADY KNOWN ON THIS TOPIC**

**WHAT THIS STUDY ADDS**

- This is the first study examining glucocorticoids and biological disease-modifying antirheumatic drug (bDMARD) treatments’ effects on PCSK9 levels in newly diagnosed DMARD-naïve rheumatoid arthritis patients.
- Our results suggest that glucocorticoid treatment increases PCSK9 levels, whereas PCSK9 levels remain, in essence, unaltered with bDMARD treatments.

**HOW THIS STUDY MIGHT AFFECT RESEARCH, PRACTICE OR POLICY**

- Our findings could provide a potential mechanistic link between glucocorticoid treatment and cardiovascular disease. These findings may have clinical implications for the prevention of cardiovascular risk.

## Introduction

 Rheumatoid arthritis (RA) is a chronic systemic inflammatory disease associated with an increased burden of atherosclerotic cardiovascular disease.^1^ Atherosclerosis is a slow, pathological process that can begin in early life and remains asymptomatic for a long period before showing clinical manifestations.^2^ Low-density lipoprotein (LDL) cholesterol is a main driver of atherosclerosis development. The key regulator of LDL metabolism is proprotein convertase subtilisin/kexin type 9 (PCSK9), which regulates circulating LDL cholesterol through the reduction of LDL receptors on the hepatic cell surface, leading to increased circulating LDL cholesterol.^3^

Decreased PCSK9 gene expression due to the loss of function mutation has been associated with low LDL cholesterol and decreased cardiovascular risk, whereas increased PCSK9 gene expression due to the gain of function mutation has shown the opposite effect.^4^

**Figure 1:**
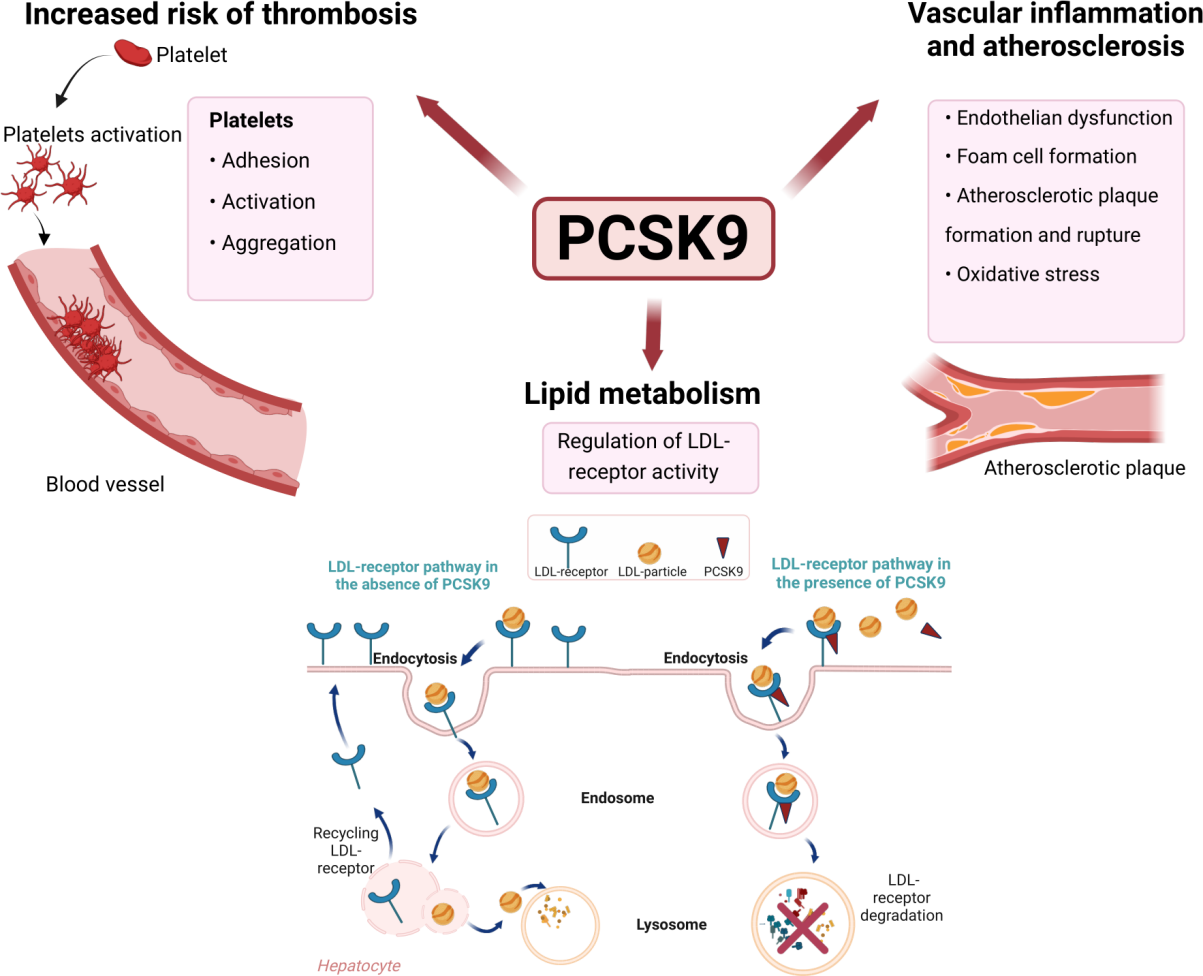
Involvement of PCSK9 in lipid metabolism, its role in increased risk of thrombosis, vascular inflammation and atherosclerosis. LDL, low-density lipoprotein; PCSK9, proprotein convertase subtilisin/kexin type 9.

In recent years, several lines of evidence have demonstrated the role of PCSK9 in the pathophysiology of cardiovascular disease, atherogenic effects and subclinical vascular changes partly independently of its effect on lipid metabolism,^5^,^7^ as illustrated in figure 1.

Experimental studies have suggested that PCSK9 plays a substantial role in every step of atherosclerotic plaque formation. PCSK9 contributes to the formation of foam cells, induces the expression of inflammatory cytokines consequently enhancing monocyte recruitment, causes inflammation in atherosclerosis and contributes to oxidative stress, which accelerates atherosclerotic development.^3^ Furthermore, PCSK9 has been suggested to contribute to the development of atherosclerotic plaque and thrombosis by promoting platelet activation, aggregation and clot formation.^5^ Also, the fraction and amount of necrotic tissue in coronary atherosclerosis has been shown to increase proportionately to serum PCSK9 independently of LDL cholesterol.^6^ Accelerated atherosclerosis, measured by an increase in intima–media thickness (IMT), has been observed in individuals with early RA after 18 months postdiagnosis compared with controls, whereas there were no significant differences in IMT between RA patients and controls at baseline.^8^

Individuals with RA have approximately a 50% higher risk of cardiovascular disease than the general population,^1^ even though their LDL-cholesterol levels are lower than or similar to those of the general population.^9 10^ The inverse relationship between cardiovascular risk and lipid levels is known as the ‘lipid paradox’ in RA.^11 12^ Systemic inflammation has a causal role in the risk of developing cardiovascular disease,^13 14^ but the underlying pathophysiological mechanisms are not fully known.

Anticitrullinated protein antibody (ACPA) and rheumatoid factor (RF) positivity have been associated with an increased risk of cardiovascular events.^15 16^ Antinuclear antibody (ANA) positivity has been shown to be more prevalent in patients with severe coronary atherosclerosis compared with those with normal coronary arteries.^17^ Although ANA positivity is most strongly associated with systemic lupus erythematosus, where more than 95% of patients test positive, it is also linked to other autoimmune disorders and is present in approximately 20% of the general population.^18^

PCSK9 levels may be influenced by factors, such as body mass index (BMI), age as well as various metabolic and genetic determinants in the general population.^19^ A cross-sectional study in axial spondyloarthritis reported that PCSK9 levels were positively associated with disease activity, higher in glucocorticoid users and lower in patients receiving tumour necrosis factor inhibitor.^20^ Furthermore, an increased risk of cardiovascular events has been reported in steroid-naïve RA patients initiating glucocorticoid treatment.^21^ In contrast, biological disease-modifying antirheumatic drug (bDMARD) use has been associated with reduced risk of cardiovascular disease and lower likelihood of new plaque formation in patients with early atherosclerosis.^22^

This raises the question, whether the atherosclerotic process accelerates in RA patients as a result of longer exposure to chronic systemic inflammation, or if RA treatments impact PCSK9 levels and, consequently, lead to an increased risk of cardiovascular disease. The 24-week primary results of the NORD-STAR trial, a four-arm randomised controlled study in newly diagnosed early RA patients with moderate-to-high baseline disease activity, showed high remission rates across all treatment groups at 24 weeks.^23^ This trial gives us the opportunity to investigate: (1) the influence of glucocorticoids and bDMARD treatments on PCSK9 and LDL-cholesterol levels, (2) whether this influence is different in the presence of autoantibodies and (3) the association between PCSK9 and LDL-cholesterol levels.

## Methods

### Study design and participants

NORD-STAR (EudraCT2011-004720-35, NCT01491815) was a multicentre, investigator-initiated blinded-assessor, phase 4, randomised controlled trial of early RA (symptom duration<24 months), conducted in Sweden, Denmark, Norway, Finland, the Netherlands, and Iceland. Newly diagnosed DMARD-naïve patients (n=812), fulfilling the 2010 American College of Rheumatology/European Alliance of Associations for Rheumatology classification criteria for RA, aged 18 years or older, with moderate to severe disease activity (disease activity score of 28 joints, based on C reactive protein (DAS28-CRP)>3.2), and with ACPA, or RF positivity, or increased C reactive protein (≥10 mg/L) or a combination of the above were enrolled. Key exclusion criteria were administration of intra-articular or parenteral glucocorticoids in the preceding 4 weeks, a dose of oral glucocorticoids (or equivalent) >7.5 mg/day or a dose change within the preceding 4 weeks, poorly controlled medical condition, such as uncontrolled diabetes, unstable heart disease and congestive heart failure. The primary NORD-STAR analysis was performed on the intention-to-treat population. The per-protocol population excluded participants with major protocol deviations as outlined in the previously published 24 weeks NORD-STAR statistical analysis plan.^23^ In this substudy, we included Swedish per-protocol patients who had sufficient volume of serum available for the PCSK9 laboratory analysis and were not using statins at baseline or at 24 weeks.

### Randomisation

Patients were randomly assigned in a 1:1:1:1 ratio, stratified by country, ACPA status and sex into one of the four treatment groups.

- Group 1 received methotrexate plus oral prednisolone (tapered from 20 to 5 mg per day within 9 weeks, discontinued at week 36).
- Group 2 received methotrexate plus certolizumab pegol (200 mg subcutaneously administered every other week (loading dose 400 mg at 0, 2 and 4 weeks).
- Group 3 received methotrexate plus abatacept (125mg subcutaneously administered every week).
- Group 4 received methotrexate plus tocilizumab (8 mg/kg intravenously administered every 4 weeks or 162 mg subcutaneously administered every week).

Oral glucocorticoids were not allowed in bDMARD treatment groups. However, short-term administration of glucocorticoids for acute medical conditions other than RA was permitted. Intra-articular glucocorticoid injections were allowed when clinically indicated, but not 4 weeks before the 24-week visit. Details have been previously published.^23 24^

### Procedures

#### Laboratory measures

Serum samples were obtained at baseline and at 24 weeks after initiation of the randomised treatment and stored at −80 °C until analysis.

PCSK9 levels were determined by a commercial ELISA kit (R&D Systems, UK) as per manufacturer’s instructions. The assay range of ELISA was 125.0–8000 pg/mL. A 100-fold dilution was performed on the samples to ensure they align with the specified range.

Analyses of total cholesterol, high-density lipoprotein (HDL) cholesterol and triglycerides were performed at Amsterdam University Medical Centres laboratory. LDL cholesterol was calculated by the laboratory using Friedewald’s formula ((LDL cholesterol) = (total cholesterol) − (HDL cholesterol) −(triglycerides)/2.2 in mmol/L).^25^ Assay ranges were as follows: total cholesterol 0.1–20.7 mmol/L, HDL cholesterol 0.08–3.88 mmol/L and triglycerides 0.1–10.0 mmol/L.

ACPA and RF were measured using the EliA (immunoglobulin G and immunoglobulin M) methods on a Phadia 250 instrument from ThermoFisher Scientific (Freiburg, Germany https://www.thermofisher.com/phadia/wo/en/our-solutions/elia-autoimmunity-solutions/rheumatoid-arthritis.html) according to suppliers’ instructions. RF positivity was defined as a concentration of greater than 5 IU/mL and ACPA positivity as a concentration of greater than 10 U/mL.

ANA test was done at local certified laboratories and results (positive or negative) were recorded in the NORD-STAR Case Report Forms.

### Statistical analysis

Baseline characteristics are presented as proportions for categorical variables, as means (SD) for normally distributed continuous variables or as medians (IQR) for non-normally distributed continuous variables.

In the first analysis, a linear regression model was used to assess the effect of RA treatments on PCSK9 levels at 24 weeks. The model included PCSK9 levels at baseline as a covariate to adjust for baseline differences. The treatment variable was included in the model, with glucocorticoid treatment as the reference for each of the three different bDMARD treatments. The same linear regression analysis was applied to assess LDL cholesterol at 24 weeks, adjusting for LDL-cholesterol levels at baseline.

In the second analysis, we combined the three bDMARD treatments into one group and conducted the same analysis.

To investigate whether the influence of glucocorticoids and bDMARD treatments on PCSK9 levels at 24 weeks was different in the presence of autoantibodies (RF, ACPA or ANA), interaction between treatment and autoantibody status was added to the linear regression model. The same analysis method was applied for LDL-cholesterol assessment.

To estimate the association between LDL cholesterol and PCSK9 over time (between baseline and 24 weeks), a linear mixed model analysis was used with LDL cholesterol as outcome. First, we analysed the relationship on average over time and, second, the relationship at baseline and at 24 weeks. In the first analysis, the treatment and time variables were included in the model. In the latter analysis, treatment and an interaction between PCSK9 and time were added to the model.

Besides crude analyses, all analyses were also adjusted for sex, age, BMI, DAS28-CRP, ACPA and RF at baseline. Normality of the residuals of all analyses was evaluated by visual inspection.

The statistical analysis was done using Stata (V.18) and SPSS (V.28). GraphPad Prism (V.10) and BioRender were used to create the figures.

The NORD-STAR trial is registered with EudraCT (2011-004720-35) and ClinicalTrials.gov (NCT01491815).

### Funding

Inger Bendix Foundation for Medical Research contributed to the funding of the cholesterol analysis for this study.

#### Role of the funding source

Funding sources of this study and the NORD-STAR investigator-initiated trial had no role in study design, collection, analysis and interpretation of data, in the writing of the report or in the decision to submit for publication.

## Results

**Figure 2:**
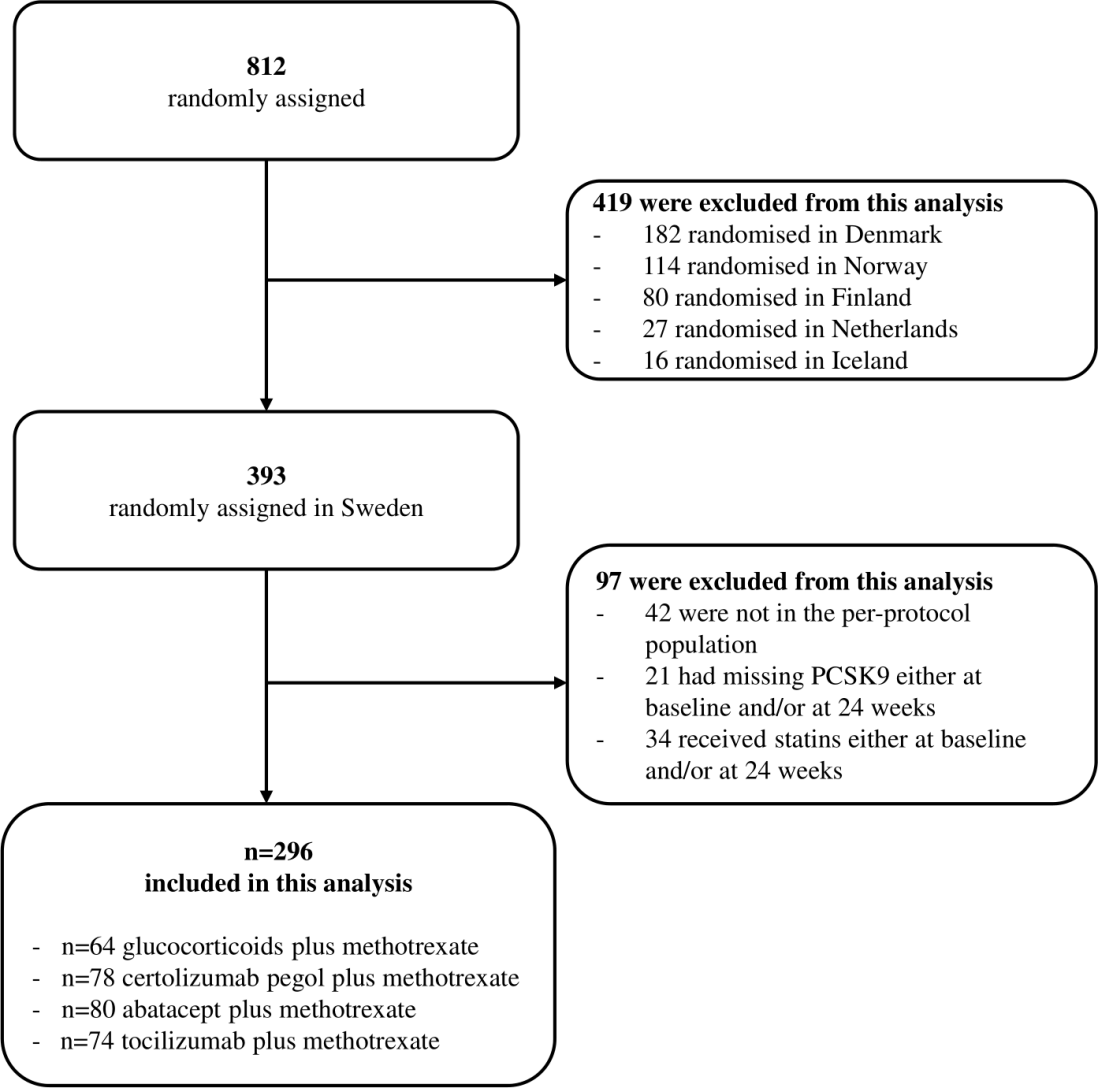
Flowchart of included patients. PCSK9, proprotein convertase subtilisin/kexin type 9.

Between December 2012 and December 2018, 393 of 812 (48%) NORD-STAR patients were randomised in Sweden, of whom 42 (11%) had major protocol violations, such as withdrawal from the study, permanent discontinuation of the randomised study medication or interruptions in treatment lasting 12 weeks or more, leading to the exclusion of the patient from the per-protocol population. Detailed information on the reasons for exclusion has been previously published.^23^ Of the 351 per-protocol patients, 21 (6%) had insufficient serum sample volume available for the PCSK9 analysis and 34 (10%) were statin users, either at baseline or at 24 weeks, and were excluded, as statins are well known to affect LDL-cholesterol levels,^26^ but also PCSK9 levels.^27^ In total, 296 Swedish patients were included in this substudy, of which 64 (22%) received glucocorticoids plus methotrexate, 78 (26%) received certolizumab pegol plus methotrexate, 80 (27%) received abatacept plus methotrexate and 74 (25%) received tocilizumab plus methotrexate (figure 2). In the glucocorticoids plus methotrexate treatment group, the daily oral glucocorticoid dosage started at 20 mg, which was tapered to 5 mg within 9 weeks. At 24 weeks, the average daily glucocorticoid dosage was 4.8 mg. The received oral glucocorticoid dosage was comparable irrespective of autoantibody status; the details are provided in online supplemental table 1.

The number of patients on glucocorticoids at baseline was higher in the tocilizumab treatment group; otherwise, baseline characteristics were well balanced, as shown in table 1.

**Table 1:** Baseline characteristics and medications of interest of patients with early RA

	Group 1: methotrexate plus glucocorticoids(n=64)*	Group 2: methotrexate plus certolizumab pegol (n=78)†	Group 3: methotrexate plus abatacept(n=80)‡	Group 4: methotrexate plus tocilizumab(n=74)§
Baseline characteristics				
Female	43/64 (67%)	54/78 (69%)	56/80 (70%)	49/74 (66%)
Age (years)	55.8 (15.1)	53.3 (16.2)	54.4 (15.5)	52.7 (12.6)
Symptom duration, days	157 (105–269)	147 (85–227)	177 (94–253)	165 (94–286)
Time since diagnosis, days	2 (0–7)	2 (0–7)	5 (0–12)	3 (0–10)
BMI, kg/m^2^	26.4 (5.2)	25.0 (4.4)	25.9 (4.4)	26.3 (5.1)
Smoking				
Current smoker	11/64 (17%)	18/78 (23%)	17/80 (21%)	20/74 (27%)
Former smoker	26/64 (41%)	24/78 (31%)	31/80 (39%)	22/74 (30%)
Non-smoker	27/64 (42%)	36/78 (46%)	32/80 (40%)	32/74 (43%)
ACPA positive	54/64 (84%)	63/78 (81%)	67/80 (84%)	66/74 (89%)
RF positive	48/64 (75%)	62/78 (80%)	66/80 (83%)	60/74 (81%)
ANA positive	19/59 (32%)	29/72 (40%)	34/79 (43%)	19/72 (26%)
CDAI score	30.2 (13.2)	30.5 (12.4)	31.5 (11.8)	27.8 (11.6)
DAS28-CRP¶	5.2 (1.2)	5.2 (1.1)	5.3 (1.0)	5.0 (1.0)
C reactive protein, mg/L	15 (5–31)	14 (4–41)	10 (5–29)	9 (5-20)
Alcohol consumption **				
Never	5/64 (8%)	12/78 (15%)	7/79 (9%)	10/73 (14%)
Less than two times a week	42/64 (66%)	46/78 (59%)	54/79 (68%)	51/73 (70%)
Two or more times a week	17/64 (27%)	20/78 (26%)	18/79 (23%)	12/73 (16%)
PCSK9 (pg/mL)	1509 (648)	1661 (888)	1611 (639)	1722 (977)
Cholesterol (mmol/L)				
Total cholesterol	5.1 (1.0)	5.0 (1.1)	5.3 (1.0)	4.9 (0.9)
HDL cholesterol	1.4 (0.3)	1.4 (0.4)	1.4 (0.3)	1.4 (0.4)
LDL cholesterol	3.2 (0.8)	3.0 (0.9)	3.3 (0.9)	3.0 (0.7)
Triglycerides (mmol/L)	1.1 (0.8–1.6)	1.1 (0.7–1.5)	1.1 (0.7–1.5)	1.0 (0.7–1.5)
Medications of interest at baseline and at 24 weeks				
Oral glucocorticoid use at baseline	1/64 (1.6%)	2/78 (2.6%)	1/80 (1.3%)	7/74 (9.5%)
Oral glucocorticoid dose at 24 weeks (mg)	4.8 (1.3)	0.1 (0.8)	0.1 (0.9)	0.4 (1.5)
Cumulative oral glucocorticoid dose (mg)	1511 (163)	32 (141)	34 (186)	95 (299)

Data are n/N (%), mean (SD) or median (IQR).

One missing BMI value was imputed with the median.

* Missing data as follows: n=5 for ANA status, n=1 for CDAI score, n=1 for C reactive protein, n=2 for total cholesterol, n=2 for HDL cholesterol, n=2 for LDL cholesterol and n=2 for triglycerides.

† Missing data as follows: n=6 for ANA status, n=2 for CDAI score, n=4 for total cholesterol, n=3 for HDL cholesterol, n=4 for LDL cholesterol and n=3 for triglycerides.

‡ Missing data as follows: n=1 for BMI, n=1 for ANA status, n=1 for alcohol consumption, n=3 for total cholesterol, n=4 for HDL cholesterol, n=4 for LDL cholesterol and n=3 for triglycerides.

§ Missing data as follows: n=2 for ANA status, n=1 for alcohol consumption, n=4 for total cholesterol, n=3 for HDL cholesterol, n=4 for LDL cholesterol and n=3 for triglycerides.

¶ DAS28-CRP was replaced with DAS28-ESR for one patient.

** The alcohol intake question in the case report forms was ‘How often do you have a drink containing alcohol?’

ACPA, anticitrullinated protein antibody; ANA, antinuclear antibody; BMI, body mass index; CDAI, clinical disease activity index; DAS28-CRP, disease activity score of 28 joints, based on C reactive protein; ESR, erythrocyte sedimentation rate; HDL, high-density lipoprotein; LDL, low-density lipoprotein; PCSK9, proprotein convertase subtilisin/kexin type 9; RA, rheumatoid arthritis; RF, rheumatoid factor.

**Figure 3:**
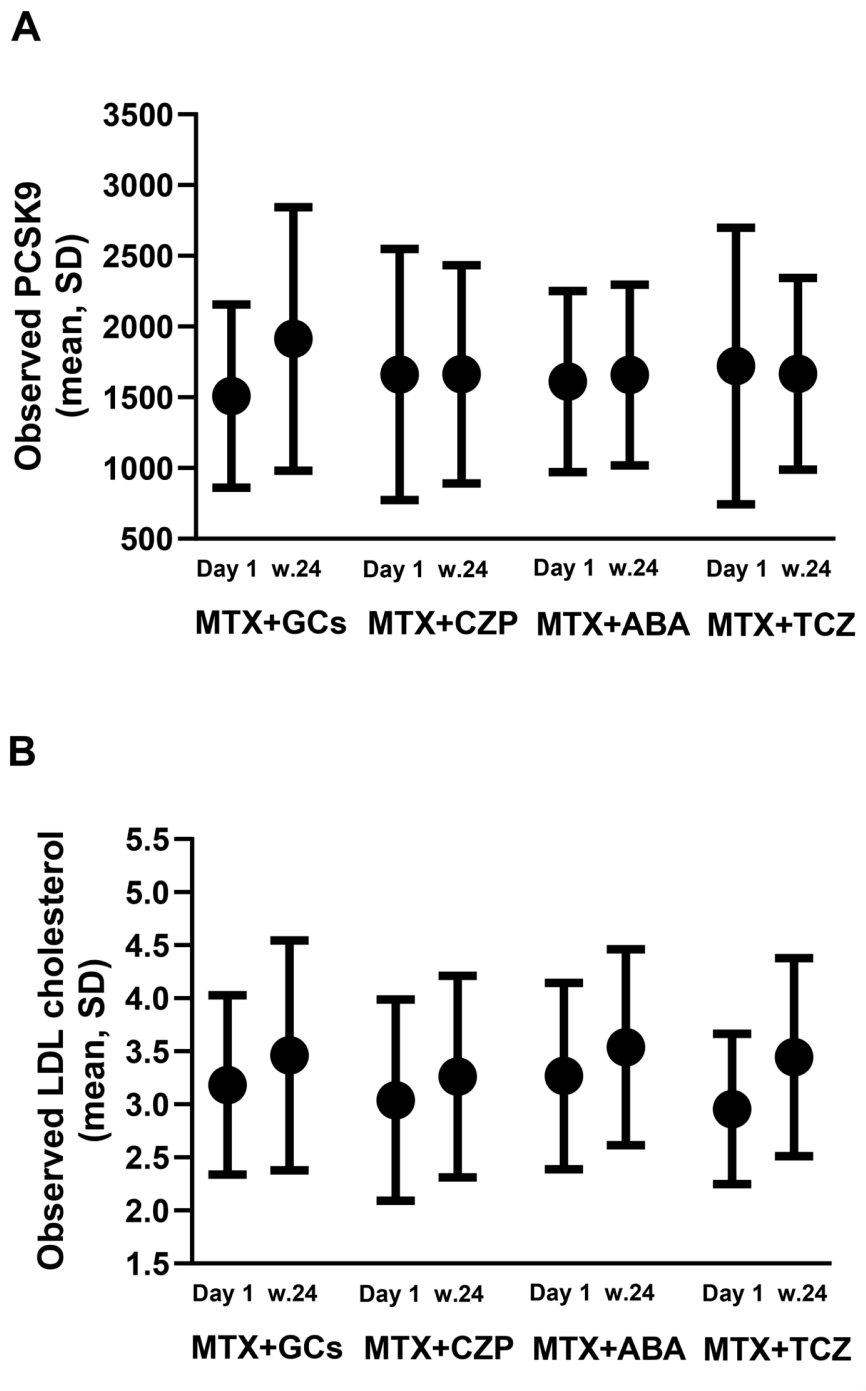
Observed PCSK9 and LDL cholesterol presented at baseline and at 24 weeks, stratified by treatment group. The ELISA assay range applied for PCSK9 analysis was 125.0–8000 pg/mL. (**A**) PCSK9 (pg/mL), presented as mean±SD. (**B**) LDL cholesterol (mmol/L), presented as mean±SD. ABA, abatacept; CZP, certolizumab pegol; GCs, glucocorticoids; LDL, low-density lipoprotein; MTX, methotrexate; PCSK9, proprotein convertase subtilisin/kexin type 9; TCZ, tocilizumab.

Figure 3 illustrates the observed PCSK9 and LDL cholesterol at baseline and at 24 weeks for four different treatment groups. After 24 weeks of RA treatment, an increase in PCSK9 levels was observed with glucocorticoid plus methotrexate treatment, while PCSK9 levels remained largely unchanged in patients treated with certolizumab pegol plus methotrexate, abatacept plus methotrexate or tocilizumab plus methotrexate. Changes in LDL cholesterol exhibited a pattern distinct from PCSK9, as it increased in all treatment groups.

Table 2 shows the crude and adjusted results of the PCSK9 and LDL-cholesterol analyses between glucocorticoid plus methotrexate treatment (reference) versus three different bDMARDs plus methotrexate treatments. At 24 weeks, PCSK9 levels were higher in patients receiving glucocorticoid plus methotrexate treatment than in those receiving certolizumab pegol, abatacept or tocilizumab (each combined with methotrexate), whereas LDL cholesterol did not show any notable differences between treatments. When biological treatments were analysed as a combined bDMARD treatment group, PCSK9 levels were higher in the glucocorticoid group compared with the combined bDMARD treatment group (adjusted difference −276.0 (95% CI −468.2 to −83.9)).

**Table 2:** Results of regression analysis investigating PCSK9 and LDL cholesterol at 24 weeks between glucocorticoid treatment versus biological treatments

	Glucocorticoids plus methotrexate	Certolizumab pegol plus methotrexate	Abatacept plus methotrexate	Tocilizumab plus methotrexate	Combined bDMARD plus methotrexate
		Difference(95% CI)	Difference(95% CI)	Difference(95% CI)	Difference(95% CI)
	**Crude analysis**				
PCSK9 at w.24	Reference	−300.5(−535.8 to −65.2)	−289.1(−522.8 to −55.4)	−317.8(−556.4 to −79.2)	−302.0(−498.5 to −105.5)
LDL cholesterol at w.24	Reference	−0.12(−0.36 to 0.11)	−0.02(−0.25 to 0.22)	0.07(−0.17 to 0.31)	−0.03(−0.22 to 0.17)
	**Adjusted analysis**				
PCSK9 at w.24	Reference	−244.5(−475.4 to −13.6)	−279.0(−507.0 to −51.0)	−305.0(−538.0 to −72.0)	−276.0−468.2 to −83.9)
LDL cholesterol at w.24	Reference	−0.07(−0.29 to 0.15)	0.03(−0.19 to 0.26)	0.11(−0.11 to 0.34)	0.02(−0.16 to 0.21)

PCSK9 is expressed in pg/mL, measured with an assay range of 125.0–8000 pg/mL. LDL cholesterol is expressed in mmol/L, calculated using Friedewald’s formula ((LDL cholesterol) = (total cholesterol) − (HDL cholesterol) −(triglycerides)/2.2 in mmol/L).^25^ Assay ranges were as follows: total cholesterol 0.1–20.7 mmol/L, HDL cholesterol 0.08–3.88 mmol/L and triglycerides 0.1–10.0 mmol/L. Combined bDMARD refers to treatment with either certolizumab pegol, abatacept or tocilizumab. Analyses were adjusted for the baseline value of the outcome variable (ie, either PCSK9 or LDL cholesterol), sex, age, BMI, DAS28-CRP, ACPA status and RF status at baseline.

ACPA, anticitrullinated protein antibody; bDMARD, biological disease-modifying antirheumatic drug; BMI, body mass index; DAS28-CRP, disease activity score of 28 joints, based on C reactive protein; HDL, high-density lipoprotein; LDL, low-density lipoprotein; PCSK9, proprotein convertase subtilisin/kexin type 9; RF, rheumatoid factor.

**Figure 4:**
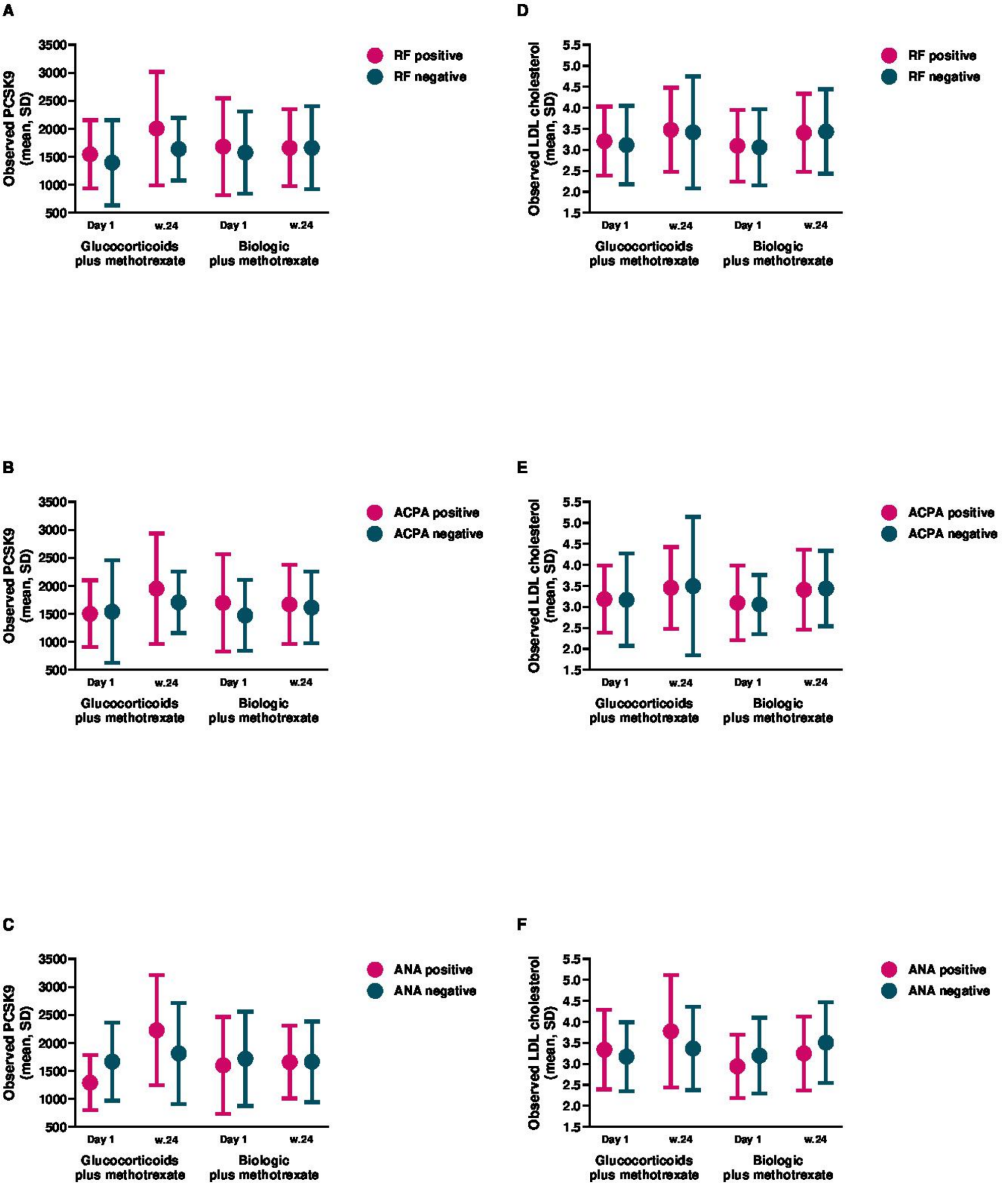
Observed PCSK9 and LDL cholesterol stratified by autoantibody status and treatment, presented as mean±SD. ACPA, anticitrullinated protein antibody; ANA, antinuclear antibody; LDL, low-density lipoprotein; PCSK9, proprotein convertase subtilisin/kexin type 9; RF, rheumatoid factor.

Figure 4 illustrates the observed PCSK9 and LDL-cholesterol levels stratified by autoantibody status at baseline and at 24 weeks. Table 3 summarises the autoantibody-stratified comparison analysis between glucocorticoid plus methotrexate treatment and the combined bDMARD treatment plus methotrexate. Overall, PCSK9 levels differed markedly in autoantibody-positive patients between glucocorticoid plus methotrexate treatment and bDMARD plus methotrexate treatment, while the PCSK9 levels were largely similar in autoantibody-negative patients. At 24 weeks, the largest difference was observed for ANA status, where PCSK9 levels were considerably higher in ANA-positive patients receiving glucocorticoid plus methotrexate treatment compared with ANA-positive patients receiving bDMARD treatment (adjusted difference −628.7 (95% CI −976.2 to −281.2)), while no substantial difference was observed for ANA-negative patients between glucocorticoids plus methotrexate versus bDMARD treatment (adjusted difference −146.7 (95% CI −389.6 to 96.2)). A similar, although less pronounced, pattern was observed for both RF and ACPA status.

**Table 3:** Results of analysis investigating the influence of autoantibody status on PCSK9 and LDL cholesterol between glucocorticoid treatment and combined biological treatment at 24 weeks

	Glucocorticoids plus methotrexate	Combined bDMARD plus methotrexate	
		Crude analysis	Adjusted analysis
		Difference (95% CI)	Difference (95% CI)
PCSK9 analysis, stratified by autoantibody status			
RF status			
positive	Reference	−387.7 (−612.3 to −163.1)	−378.7 (−597.7 to −159.7)
negative	Reference	−35.0 (−440.1 to 370.1)	56.0 (−338.4 to 450.4)
ACPA status			
positive	Reference	−344.9 (−559.4 to −130.5)	−336.7 (−544.8 to −128.6)
negative	Reference	−71.0 (−567.8 to 425.7)	62.0 (−427.6 to 551.6)
ANA status			
positive	Reference	−671.0 (−1024.3 to −317.8)	−628.7 (−976.2 to −281.2)
negative	Reference	−165.2 (−412.8 to 82.4)	−146.7 (−389.6 to 96.2)
LDL-cholesterol analysis, stratified by autoantibody status			
RF status			
positive	Reference	−0.03 (−0.26 to 0.20)	0.04 (−0.18 to 0.26)
negative	Reference	−0.01 (−0.41 to 0.39)	−0.03 (−0.41 to 0.35)
ACPA status			
positive	Reference	−0.02 (−0.24 to 0.19)	0.03 (−0.18 to 0.23)
negative	Reference	−0.03 (−0.53 to 0.47)	0.00 (−0.48 to 0.48)
ANA status			
positive	Reference	−0.24 (−0.62 to 0.13)	−0.24 (−0.59 to 0.12)
negative	Reference	0.07 (−0.18 to 0.33)	0.14 (−0.10 to 0.38)

Definitions for all other variables, including adjustment variables, are provided in the footnote of table 2.

ACPA, anticitrullinated protein antibody; ANA, antinuclear antibody; bDMARD, biological disease-modifying antirheumatic drug; LDL, low-density lipoprotein; PCSK9, proprotein convertase subtilisin/kexin type 9; RF, rheumatoid factor.

Autoantibody status did not exert a discernible effect on LDL-cholesterol levels.

The autoantibody-stratified analyses between glucocorticoid treatment versus each of the three biological treatments showed comparable results (online supplemental figure 1 and tables 2, 3).

The adjusted linear mixed model analyses investigating the association between PCSK9 and LDL cholesterol showed that, on average, across two time points (baseline and week 24), PCSK9 was associated with LDL cholesterol. The interaction analysis with time revealed that the association between PCSK9 and LDL cholesterol was strongest at baseline but weakened by week 24 (table 4).

**Table 4:** Results of analysis investigating the association between LDL cholesterol and PCSK9 over time

	Crude analysis	Adjusted analysis
PCSK9 over time	0.14 (0.07–0.22)	0.12 (0.05–0.20)
PCSK9 at baseline	0.17 (0.08–0.27)	0.15 (0.06–0.24)
PCSK9 at 24 weeks	0.11 (0.01–0.21)	0.09 (−0.01–0.19)

Definitions for variables, including adjustment variables, are provided in the footnote of table 2.

LDL, low-density lipoprotein; PCSK9, proprotein convertase subtilisin/kexin type 9.

Descriptive analysis revealed that the largest decrease in total cholesterol/HDL-cholesterol ratio was observed in the glucocorticoids plus methotrexate treatment (online supplemental table 4). Although the association between PCSK9 and total cholesterol/HDL-cholesterol ratio was strong at baseline, it diminished by week 24 (online supplemental table 5).

For all analyses, the residuals were more or less normally distributed, indicating that no transformations were necessary (online supplemental tables 6–8).

## Discussion

This is the first study to investigate the effects of glucocorticoid treatment and bDMARD treatments on PCSK9 levels in newly diagnosed DMARD-naïve RA patients. After 24 weeks of treatment, we observed an increase in PCSK9 among participants assigned to the glucocorticoid plus methotrexate treatment, whereas PCSK9 did not appear to increase within the three patient groups randomised to bDMARD plus methotrexate treatments.

Glucocorticoid treatment has been associated with an increased risk of cardiovascular events,^21^ including venous thromboembolism.^13 28^ In contrast, a notable reduction in venous thromboembolism has been observed with PCSK9 inhibitor treatment,^29^ suggesting PCSK9’s potential role in the heightened risk of venous thromboembolism with glucocorticoid treatment.

Furthermore, initiation of glucocorticoid treatment in steroid-naïve RA patients has been linked to an increased risk of cardiovascular events, with risk thresholds depending on daily dose, cumulative dose and duration of treatment.^21^ One study with a duration of 2 years found that a low dose of prednisolone (5 mg/day) may have a favourable balance of benefits of controlling the inflammation and the potential harm.^30^ Another study with longer follow-up duration (mean 8.7 years) has shown that daily prednisolone doses≥5 mg increase the risk of major adverse cardiovascular events incidence from the third year, whereas very low prednisolone doses (<5 mg daily) did not seem to confer substantial cardiovascular risk.^31^

One study found that glucocorticoid use, especially daily doses greater than 7.5 mg or cumulative doses exceeding 7000 mg, increased the risk of cardiovascular events in RF-positive patients, whereas no increased risk was observed in RF-negative patients. However, there were significant differences between RF-positive and RF-negative patients in terms of cumulative glucocorticoid doses and duration of exposure.^32^

In our study, PCSK9 increased with glucocorticoid treatment irrespective of autoantibody status; however, the increase was more pronounced in RF-, ACPA- and ANA-positive patients when compared with bDMARD plus methotrexate treatment.

Considering the crucial role of PCSK9 in regulating LDL-cholesterol homeostasis, we examined LDL cholesterol in our study. Changes in LDL cholesterol exhibited a pattern distinct from PCSK9, as it increased in all treatment groups. LDL cholesterol decreased prior to RA diagnosis.^9^ The general increase in LDL cholesterol seen in all treatment groups can be explained by the abnormally elevated LDL catabolism in active RA^33 34^ that normalises with the control of inflammation.

We investigated the relationship between PCSK9 and LDL cholesterol. While PCSK9 was highly correlated with LDL cholesterol at baseline, this correlation attenuated by 24 weeks. PCSK9 and LDL cholesterol may be directly related because PCSK9 induces the degradation of hepatic LDL receptors; however, these two proteins are not invariably correlated.^35^ PCSK9 expression is primarily controlled by sterol regulatory element-binding protein 2 (SREBP2), hepatocyte nuclear factor 1-α, forkhead box O3 and Sirtuin 6.^36^

Administration of statins to lower elevated levels of LDL cholesterol is the cornerstone of therapy to reduce the risk of cardiovascular events in patients requiring clinical prevention.^26^

Treatment with statins lowers plasma levels of LDL cholesterol while concurrently elevating circulating PCSK9 through inhibition of the 3-hydroxy 3-methylglutaryl-CoA (HMG-CoA) reductase enzyme.^37^ Inhibition of HMG-CoA reductase decreases hepatic intracellular cholesterol and leads to increased SREBP2 activity, which promotes simultaneous production of LDL receptor as well as PCSK9 transcription.^37^ Therefore, the statin therapy leads to decreased LDL cholesterol as well as increased circulating PCSK9.

Administering statins to lower elevated levels of LDL cholesterol has been observed to reduce major cardiovascular event risk by 34% with 40 mg atorvastatin compared with placebo in RA patients.^13^

The question arises as to why only glucocorticoid treatment poses a greater risk for cardiovascular events than statin treatment, whereas both are associated with increased PCSK9 levels (even more with statins).

One possible explanation could be that, in addition to lowering LDL cholesterol, statin therapy may exert pleiotropic (anti-inflammatory) effects on many components involved in cardiovascular disease and atherosclerosis, such as plaque thrombogenicity, endothelial function, cellular migration and thrombotic predisposition^38^ that might be absent with glucocorticoid treatment.

A previous study found that PCSK9 was independently correlated with platelet reactivity^39^ in individuals not receiving statin therapy. PCSK9 directly enhances platelet activation and promotes in vivo thrombosis through its binding to platelet CD36 as well as enhancing P-selectin release from α-granules.^40^ On platelets, P-selectin influences the size and stability of platelet aggregates,^41^ which may play a crucial role in blood clot formation and thrombosis. Statins have shown to have some additional inhibitory effects on platelet aggregation and activation beyond lipid-independent mechanisms,^42^ potentially protecting against cardiovascular disease despite elevated levels of PCSK9. One study has shown that high-dose statins can markedly reduce soluble P-selectin concentrations^43^ that could be a part of the statins' pleiotropic effect on platelet activation and endothelial function. Furthermore, a decrease in platelet reactivity has been shown with PCSK9 inhibition treatment,^44^ suggesting that PCSK9 inhibitors as well as aspirin may reduce the risk of cardiovascular disease by counteracting the enhancing effects of PCSK9 on platelet reactivity.

Since glucocorticoid treatment is usually not combined with concomitant PCSK9 inhibitor or aspirin that prevents platelets hyperactivity and may lack the pleiotropic effects of statins, the increase in PCSK9 could explain the higher risk of cardiovascular events associated with glucocorticoid treatment.

Our study has some limitations, including the relatively small sample size. Since RF and ACPA are present in the majority of individuals with RA, our autoantibody-negative group was limited in size. We had no knowledge of fasting state, which might have influenced PCSK9.^45^ However, fasting status would likely have affected all treatment groups similarly.

Our study’s strength lies in its inclusion of newly diagnosed patients who were randomly assigned to one of the four treatment groups. Furthermore, serum samples were collected prior to the initiation of randomised treatment and again at 24 weeks. Centralised laboratory analyses of serum samples were used for the assessment of PCSK9, RF status, ACPA status and cholesterol.

In summary, our study showed that glucocorticoid treatment was associated with increased PCSK9 at 24 weeks. When compared with the bDMARD treatment, these increases were more pronounced in autoantibody-positive patients. Changes in LDL cholesterol exhibited a pattern distinct from PCSK9, as it increased in all treatment groups. Our data could provide a potential mechanistic link between glucocorticoid treatment and cardiovascular disease.

## Supplementary material

10.1136/rmdopen-2024-005129 online supplemental file 1

## Data Availability

No data are available.
