# Supplementary material for: Glucocorticoid treatment in early rheumatoid arthritis is independently associated with increased PCSK9 levels: data from a randomised controlled trial
Source: RMD Open. 2025 Jun 5;11(2):e005129. doi: 10.1136/rmdopen-2024-005129 (PMC12161332; doi:10.1136/rmdopen-2024-005129)
Supplement: online supplemental file 1 [file rmdopen-11-2-s001.docx]

SUPPLEMENTARY APPENDIX

[**Supplementary figures** 2](#_Toc195544655)

[Figure S1: Observed proprotein convertase subtilisin/kexin type 9 (PCSK9) and LDL cholesterol stratified by autoantibody status and treatment. 2](#_Toc195544656)

[**Supplementary tables** 3](#_Toc195544657)

[Table S1. Oral glucocorticoid dosage among autoantibody positive and negative patients in the glucocorticoid plus methotrexate treatment group. 3](#_Toc195544658)

[Table S2. Crude results of analysis investigating the influence of autoantibody status on PCSK9 and LDL-cholesterol between in glucocorticoid treatment versus three different biologic treatments at 24 weeks. 4](#_Toc195544659)

[Table S3: Adjusted results of analysis investigating the influence of autoantibody status on PCSK9 and LDL-cholesterol between in glucocorticoid treatment versus three different biologic treatments at 24 weeks. 6](#_Toc195544660)

[Table S4. Descriptive statistics of total cholesterol/HDL cholesterol ratio at baseline and at 24 weeks, stratified by treatment group. 8](#_Toc195544661)

[Table S5. Results of analysis investigating the association between total cholesterol/HDL cholesterol ratio and PCSK9 over time. 8](#_Toc195544662)

[Table S6. Residuals from PCSK9 and LDL Cholesterol Linear Regression Analysis of Table 2. 9](#_Toc195544663)

[Table S7. Residuals from PCSK9 and LDL Cholesterol Linear Regression Analysis with Autoantibody Stratification of Table 3. 11](#_Toc195544664)

[Table S8. Residuals from Mixed Models Analysis of Table 4. 14](#_Toc195544665)

# **Supplementary figures**

### Figure S1: Observed proprotein convertase subtilisin/kexin type 9 (PCSK9) and LDL cholesterol stratified by autoantibody status and treatment.


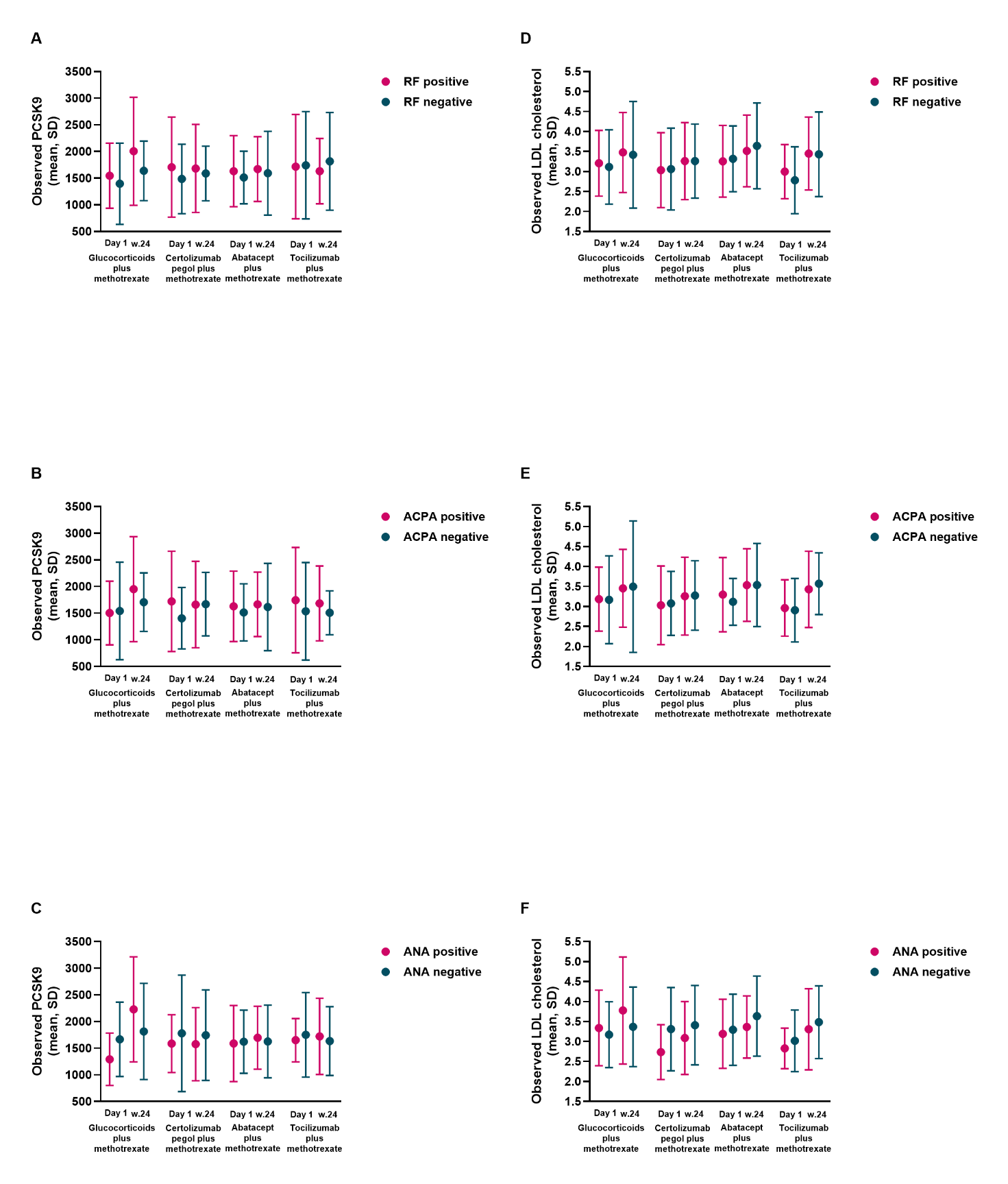


# **Supplementary tables**

### Table S1. Oral glucocorticoid dosage among autoantibody positive and negative patients in the glucocorticoid plus methotrexate treatment group.

|  | **Oral glucocorticoids dosage (mg)** | | | | | |
| --- | --- | --- | --- | --- | --- | --- |
|  | **positive** | | | **negative** | | |
|  | n (%) | GCs dose at 24 weeks (mg) | Cumulative GCs dose (mg) | n (%) | GCs dose at 24 weeks (mg) | Cumulative GCs dose (mg) |
| RF status | 48/64 (75%) | 4.9 (1.1) | 1524 (154) | 16/64 (25%) | 4.5 (1.6) | 1469 (185) |
| ACPA status | 54/64 (84%) | 4.8 (1.3) | 1506 (173) | 10/64 (16%) | 5.0 (1.2) | 1535 (92) |
| ANA status | 19/59 (32%) | 5.1 (1.0) | 1555 (155) | 40/59 (68%) | 4.7 (1.4) | 1490 (173) |

Values are mean (SD). ACPA=anti-citrullinated protein antibody. ANA=antinuclear antibody. GCs=glucocorticoids. RF=rheumatoid factor. SD=Standard deviation.

### Table S2. Crude results of analysis investigating the influence of autoantibody status on PCSK9 and LDL-cholesterol between in glucocorticoid treatment versus three different biologic treatments at 24 weeks.

|  | **Glucocorticoids plus methotrexate** | **Certolizumab pegol plus methotrexate** | **Abatacept plus methotrexate** | **Tocilizumab plus methotrexate** |
| --- | --- | --- | --- | --- |
|  |  | Difference (95% CI) | Difference (95% CI) | Difference (95% CI) |
| **Crude PCSK9 analysis, stratified by autoantibody status** | | | | |
| **RF status** |  |  |  |  |
| positive | Reference | -374.7 (-643.2 to -106.2) | -361.1 (-625.7 to -96.4) | -430.0 (-700.5 to -159.4) |
| negative | Reference | -79.5 (-572.5 to 413.5) | -83.7 (-594.1 to 426.6) | 65.2 (-446.2 to 576.6) |
| **ACPA status** |  |  |  |  |
| positive | Reference | -362.6 (-622.9 to -102.4) | -327.4 (-583.4 to -71.4) | -346.7 (-604.4 to -89.0) |
| negative | Reference | 7.3 (-563.6 to 578.2) | -83.2 (-671.2 to 504.9) | -197.6 (-860.7 to 465.6) |
| **ANA status** |  |  |  |  |
| positive | Reference | -749.2 (-1160.4 to -338.0) | -629.4 (-1028.5 to -230.2) | -625.1 (-1077.4 to -172.9) |
| negative | Reference | -105.5 (-411.0 to 199.9) | -172.3 (-474.2 to 129.7) | -207.5 (-498.6 to 83.6) |
| **Crude LDL-cholesterol analysis, stratified by autoantibody status** | | | | |
| **RF status** |  |  |  |  |
| positive | Reference | -0.12 (-0.39 to 0.16) | -0.03 (-0.30 to 0.24) | 0.07 (-0.21 to 0.34) |
| negative | Reference | -0.15 (-0.64 to 0.35) | 0.04 (-0.46 to 0.55) | 0.09 (-0.42 to 0.59) |
| **ACPA status** |  |  |  |  |
| positive | Reference | -0.11 (-0.37 to 0.15) | -0.03 (-0.29 to 0.22) | 0.07 (-0.19 to 0.33) |
| negative | Reference | -0.19 (-0.77 to 0.39) | 0.07 (-0.52 to 0.66) | 0.10 (-0.57 to 0.78) |
| **ANA status** |  |  |  |  |
| positive | Reference | -0.23 (-0.66 to 0.20) | -0.32 (-0.74 to 0.09) | -0.10 (-0.57 to 0.37) |
| negative | Reference | -0.09 (-0.40 to 0.22) | 0.15 (-0.16 to 0.46) | 0.15 (-0.15 to 0.45) |

ACPA=anti-citrullinated protein antibody. ANA=antinuclear antibody. LDL=low-density lipoprotein. RF=rheumatoid factor. PCSK9=proprotein convertase subtilisin/kexin type 9 serine protease. PCSK9 is expressed in pg/mL, measured with an assay range of 125.0–8000 pg/mL. LDL cholesterol is expressed in mmol/L, calculated using the Friedewald’s formula ([LDL cholesterol] = [total cholesterol] - [HDL cholesterol] -[triglycerides]/2.2 in mmol/L) [25]. Assay ranges were as follows: total cholesterol 0.1–20.7 mmol/L, HDL cholesterol 0.08–3.88 mmol/L, and triglycerides 0.1–10.0 mmol/L.

### Table S3: Adjusted results of analysis investigating the influence of autoantibody status on PCSK9 and LDL-cholesterol between in glucocorticoid treatment versus three different biologic treatments at 24 weeks.

|  | **Glucocorticoids plus methotrexate** | **Certolizumab pegol plus methotrexate** | **Abatacept plus methotrexate** | **Tocilizumab plus methotrexate** |
| --- | --- | --- | --- | --- |
|  |  | Difference (95% CI) | Difference (95% CI) | Difference (95% CI) |
| **Adjusted PCSK9 analysis, stratified by autoantibody status** | | | | |
| **RF status** |  |  |  |  |
| positive | Reference | -330.8 (-593.0 to -68.7) | -378.4 (-635.1 to -121.8) | -433.4 (-698.6 to -168.1) |
| negative | Reference | 10.6 (-468.4 to 489.6) | 40.5 (-456.5 to 537.5) | 133.3 (-372.3 to 638.9) |
| **ACPA status** |  |  |  |  |
| positive | Reference | -320.1 (-573.3 to -66.8) | -332.7 (-581.1 to -84.3) | -357.2 (-608.5 to -105.8) |
| negative | Reference | 140.2 (-419.2 to 699.6) | 17.9 (-555.9 to 591.8) | -11.1 (-670.7 to 648.5) |
| **ANA status** |  |  |  |  |
| positive | Reference | -673.0 (-1076.8 to 269.3) | -604.5 (-997.1 to -212.0**)** | -590.4 (-1036.2 to -144.6) |
| negative | Reference | -56.8 (-356.9 to 243.3) | -161.2 (-456.8 to 134.5) | -206.9 (-492.3 to 78.5) |
| **Adjusted LDL-cholesterol analysis, stratified by autoantibody status** | | | | |
| **RF status** |  |  |  |  |
| positive | Reference | -0.03 (-0.29 to 0.22) | 0.03 (-0.23 to 0.28) | 0.15 (-0.11 to 0.41) |
| negative | Reference | -0.19 (-0.66 to 0.27) | 0.10 (-0.38 to 0.58) | -0.01 (-0.49 to 0.48) |
| **ACPA status** |  |  |  |  |
| positive | Reference | -0.05 (-0.29 to 0.20) | 0.01 (-0.23 to 0.25) | 0.12 (-0.13 to 0.37) |
| negative | Reference | -0.17 (-0.72 to 0.38) | 0.14 (-0.42 to 0.70) | 0.07 (-0.57 to 0.71) |
| **ANA status** |  |  |  |  |
| positive | Reference | -0.27 (-0.67 to 0.14) | -0.31 (-0.71 to 0.08) | -0.04 (-0.49 to 0.41) |
| negative | Reference | 0.00 (-0.30 to 0.30) | 0.22 (-0.08 to 0.51) | 0.19 (-0.09 to 0.47) |

Definitions for variables are provided in the footnote of Table S2. Analyses were adjusted for the baseline value of the outcome variable (i.e. either PCSK9 or LDL cholesterol), sex, age, BMI, DAS28-CRP, ACPA status, and RF status at baseline.

### Table S4. Descriptive statistics of total cholesterol/HDL cholesterol ratio at baseline and at 24 weeks, stratified by treatment group.

|  | **Glucocorticoids plus methotrexate** | **Certolizumab pegol plus methotrexate** | **Abatacept plus methotrexate** | **Tocilizumab plus methotrexate** |
| --- | --- | --- | --- | --- |
| Total cholesterol/HDL ratio at baseline | 3.8 (1.0) | 3.7 (1.2) | 3.8 (1.0) | 3.6 (0.8) |
| Total cholesterol/HDL ratio at 24 weeks | 3.5 (0.9) | 3.5 (1.1) | 3.7 (1.1) | 3.7 (0.9) |

Data are mean (SD). HDL=high-density lipoprotein. Total cholesterol/HDL ratio is expressed in mmol/L. Assay ranges were as follows: total cholesterol 0.1–20.7 mmol/L, and HDL cholesterol 0.08–3.88 mmol/L.

### Table S5. Results of analysis investigating the association between total cholesterol/HDL cholesterol ratio and PCSK9 over time.

|  | **Crude analysis** | **Adjusted analysis** |
| --- | --- | --- |
| PCSK9 over time | 0.11 (0.03 to 0.18) | 0.09 (0.01 to 0.17) |
| PCSK9 at baseline | 0.15 (0.05 to 0.24) | 0.13 (0.04 to 0.23) |
| PCSK9 at 24 weeks | 0.05 (-0.05 to 0.16) | 0.03 (-0.07 to 0.13) |

Definitions for variables are provided in the footnote of Table S2. Total cholesterol/HDL ratio is expressed in mmol/L. Treatment was added to the crude model. Analyses were adjusted for treatment, sex, age, BMI, DAS28-CRP, ACPA status, and RF status at baseline.

### Table S6. Residuals from PCSK9 and LDL Cholesterol Linear Regression Analysis of Table 2.

| **Residuals from Crude Linear Regression of PCSK9 with 4 Treatment Groups (glucocorticoids and three biologics)** | **Residuals from Adjusted Linear Regression of PCSK9 with 4 Treatment Groups (glucocorticoids and three biologics)** |
| --- | --- |
| 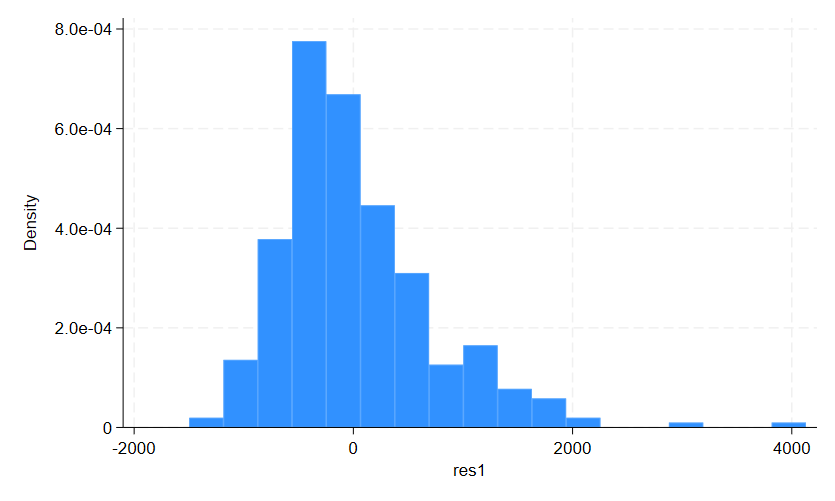 | 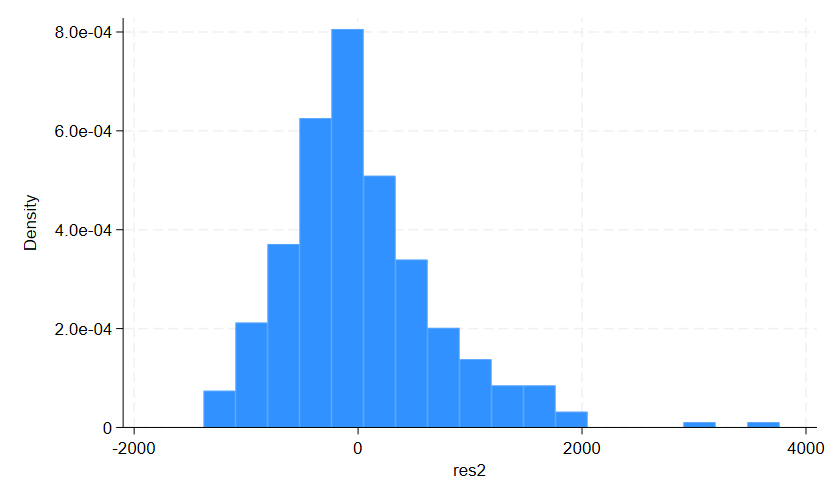 |
| **Residuals from Crude Linear Regression of PCSK9 with 2 Groups (glucocorticoids and bDMARD)** | **Residuals from Adjusted Linear Regression of PCSK9 with 2 Groups (glucocorticoids and bDMARD)** |
| 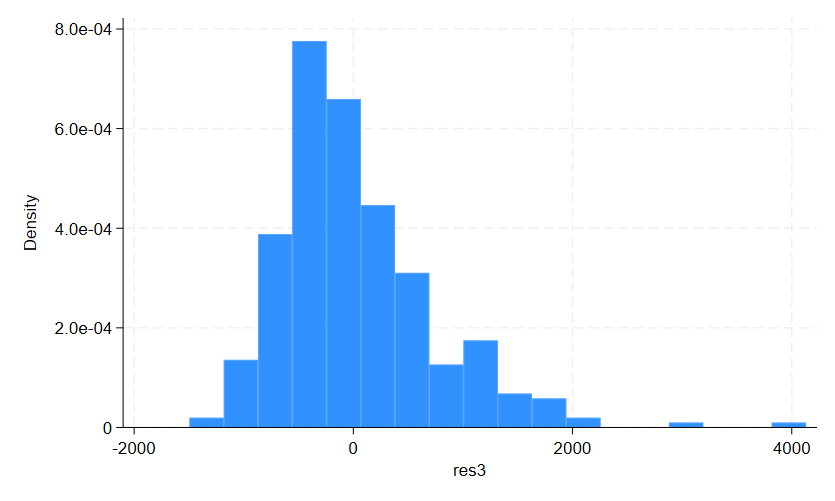 | 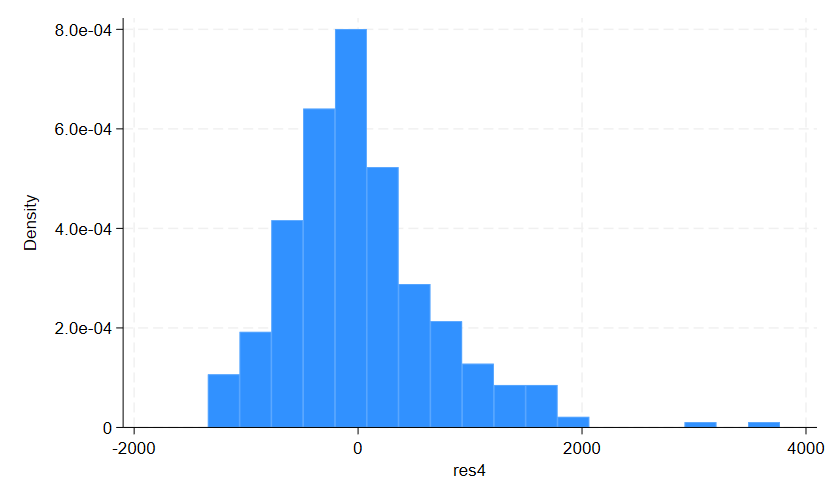 |
| **Residuals from Crude Linear Regression of LDL cholesterol with 4 Treatment Groups (glucocorticoids and three biologics)** | **Residuals from Adjusted Linear Regression of LDL cholesterol with 4 Treatment Groups (glucocorticoids and three biologics)** |
| 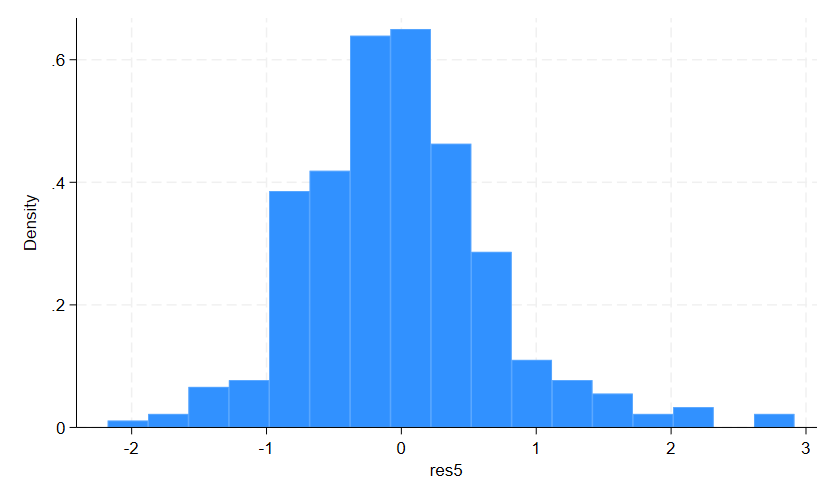 | 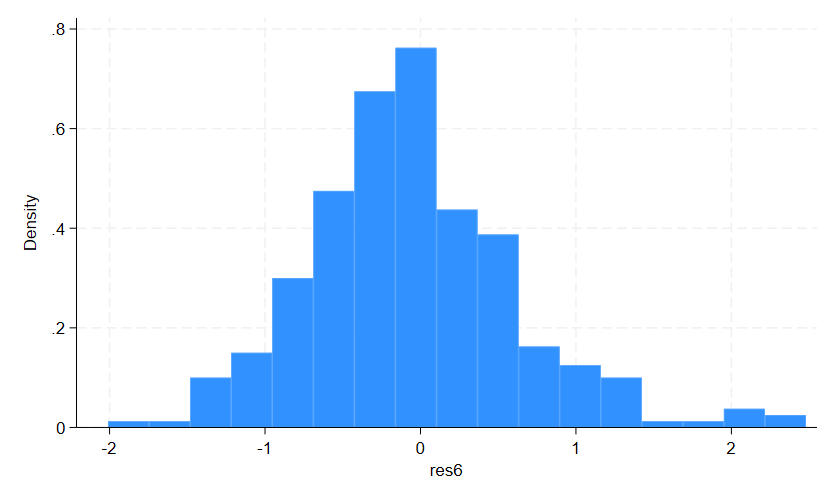 |
| **Residuals from Crude Linear Regression of LDL cholesterol with 2 Groups (glucocorticoids and bDMARD)** | **Residuals from Adjusted Linear Regression of LDL cholesterol with 2 Groups (glucocorticoids and bDMARD)** |
| 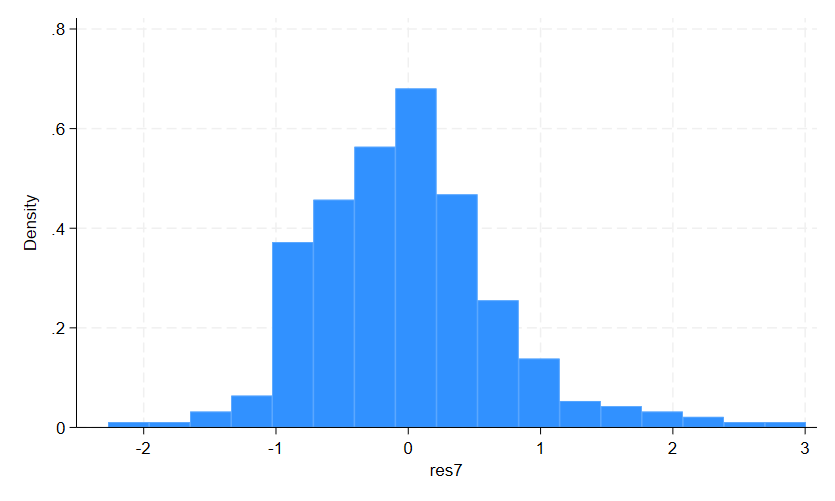 | 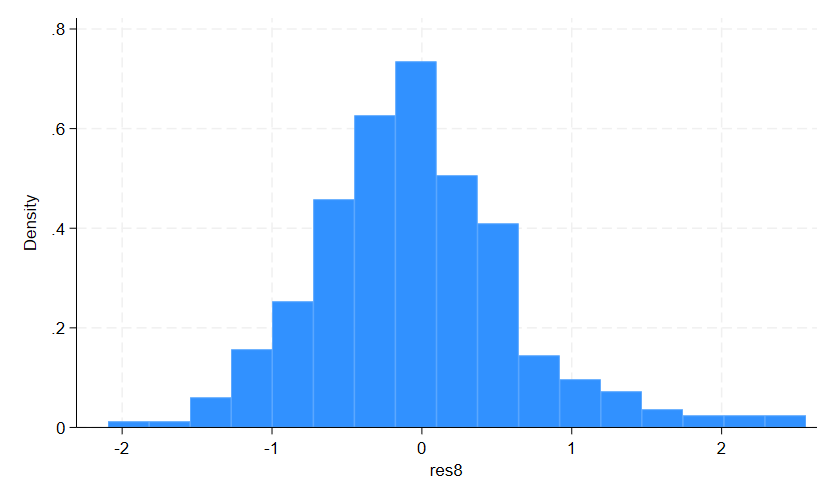 |

### Table S7. Residuals from PCSK9 and LDL Cholesterol Linear Regression Analysis with Autoantibody Stratification of Table 3.

| **Residuals from Crude Linear Regression of PCSK9 with RF Interaction** | **Residuals from Adjusted Linear Regression of PCSK9 with RF Interaction** |
| --- | --- |
| 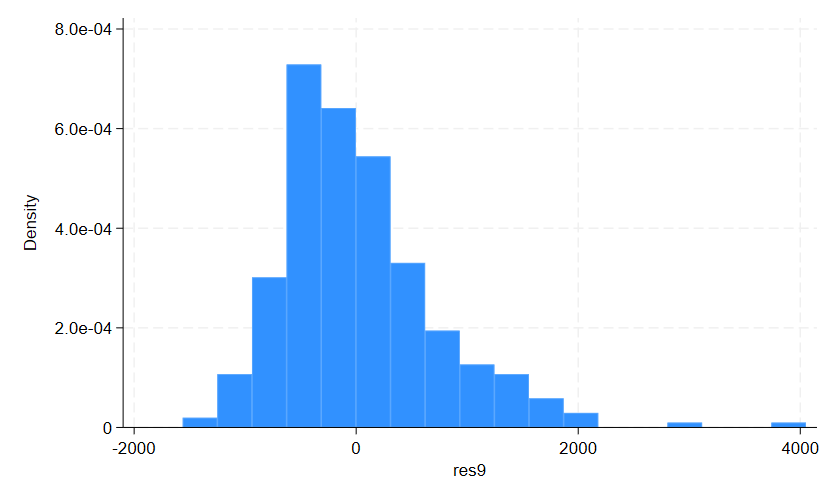 | 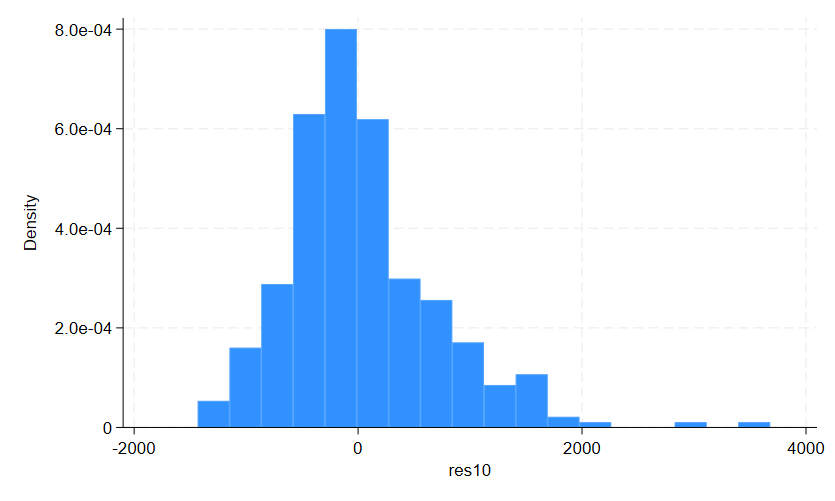 |
| **Residuals from Crude Linear Regression of PCSK9 with ACPA Interaction** | **Residuals from Adjusted Linear Regression of PCSK9 with ACPA Interaction** |
| 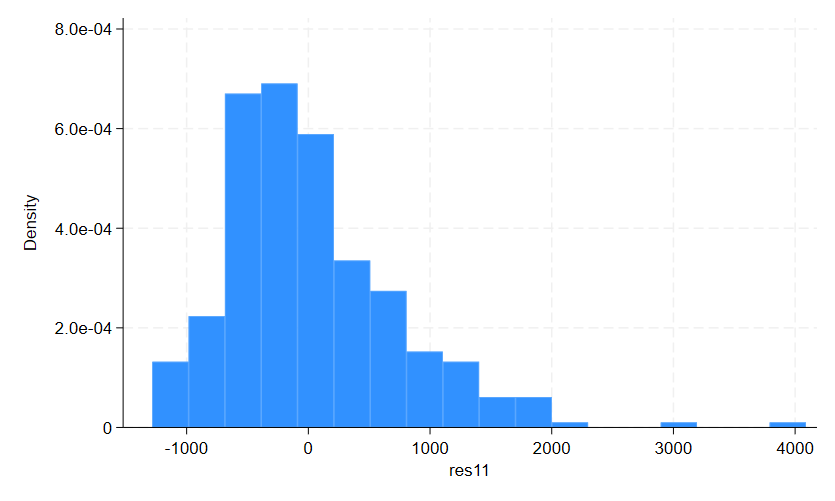 | 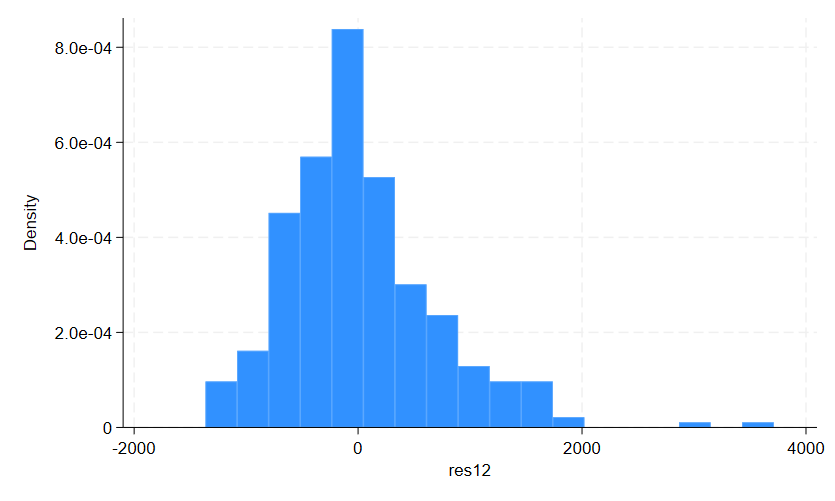 |
| **Residuals from Crude Linear Regression of PCSK9 with ANA Interaction** | **Residuals from Adjusted Linear Regression of PCSK9 with ANA Interaction** |
| **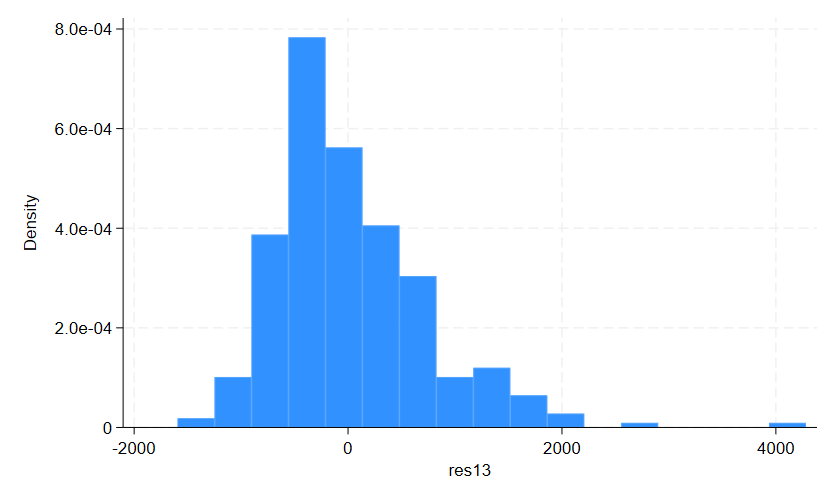** | **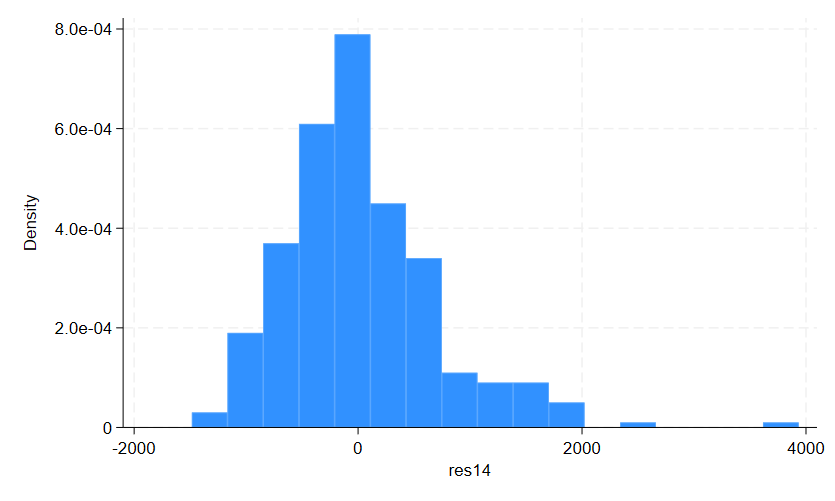** |
| **Residuals from Crude Linear Regression of LDL Cholesterol with RF Interaction** | **Residuals from Adjusted Linear Regression of LDL Cholesterol with RF Interaction** |
| 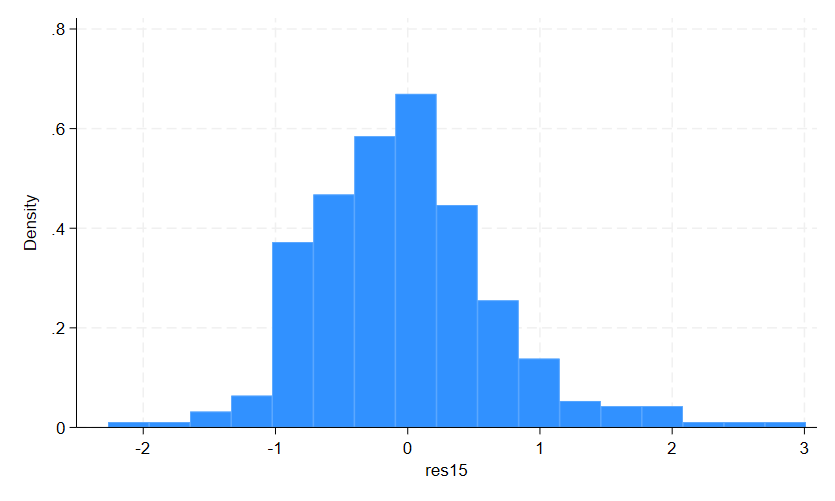 | 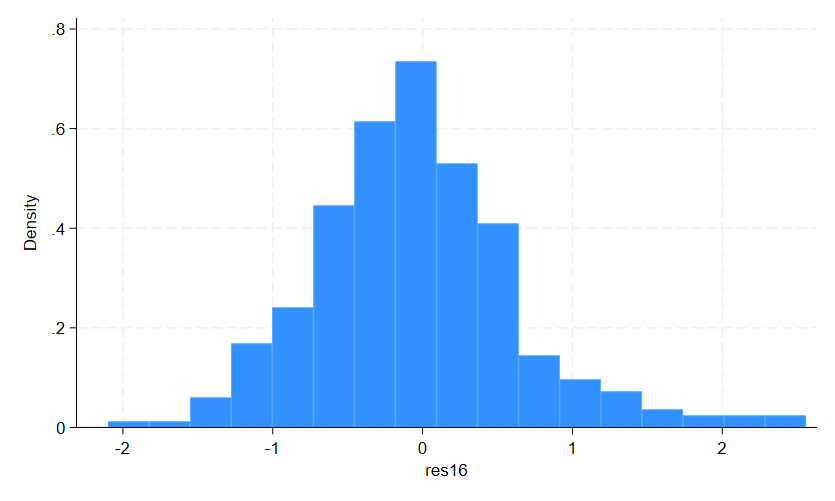 |
| **Residuals from Crude Linear Regression of LDL Cholesterol with ACPA Interaction** | **Residuals from Adjusted Linear Regression of LDL Cholesterol with ACPA Interaction** |
| 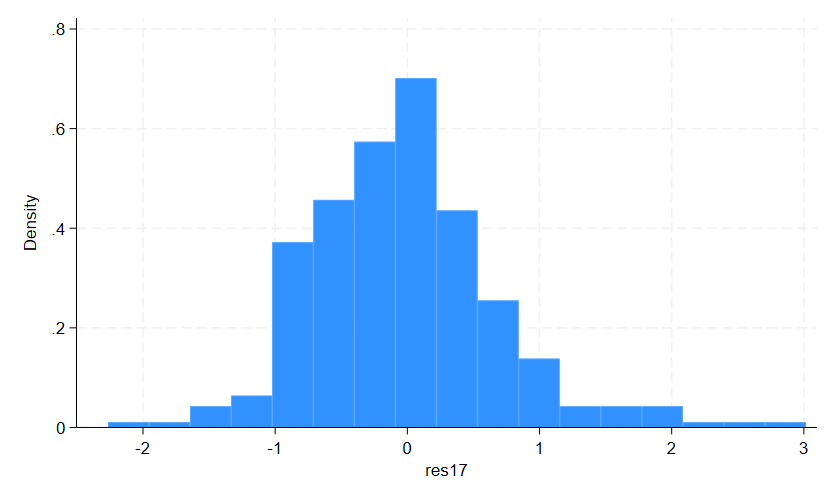 | 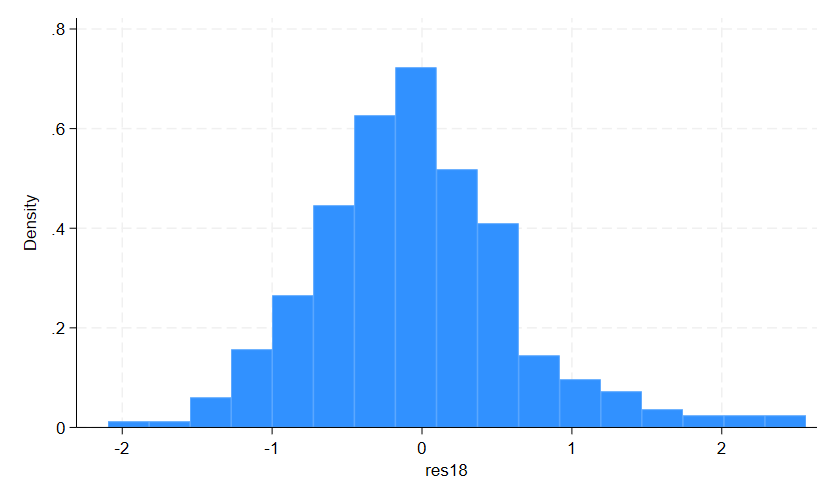 |
| **Residuals from Crude Linear Regression of LDL Cholesterol with ANA Interaction** | **Residuals from Adjusted Linear Regression of LDL Cholesterol with ANA Interaction** |
| 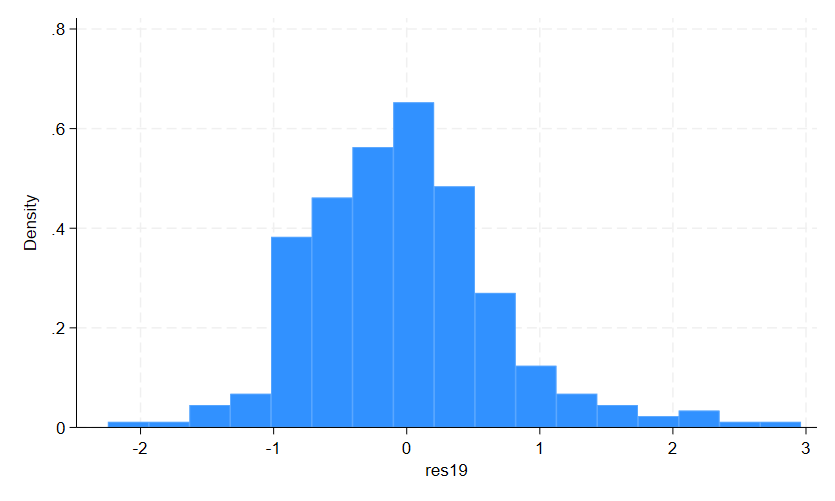 | 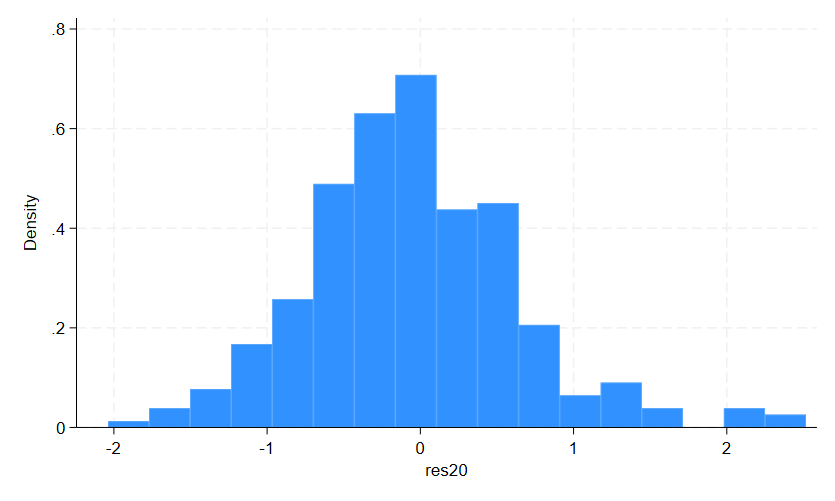 |

### Table S8. Residuals from Mixed Models Analysis of Table 4.

| **Residuals from the Crude Mixed Model Analysis over Time** | **Residuals from the Adjusted Mixed Model Analysis over Time** |
| --- | --- |
| 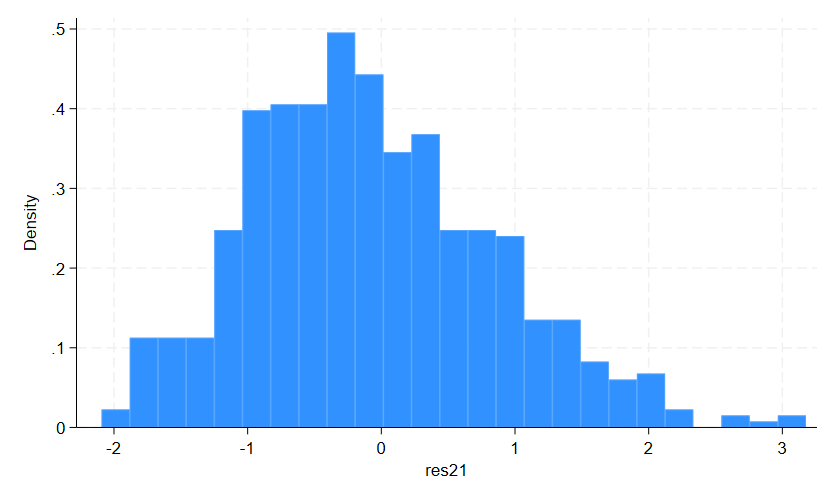 | 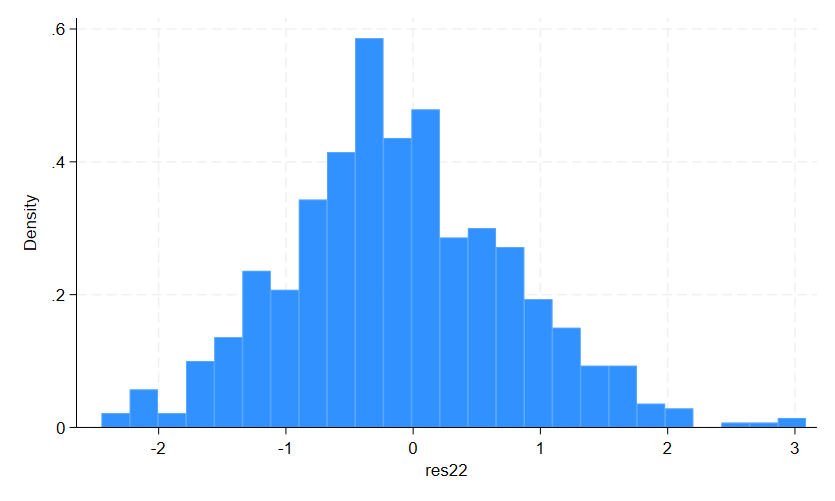 |
| **Residuals from the Crude Mixed Model Analysis with Time Interaction** | **Residuals from the Adjusted Mixed Model Analysis with Time Interaction** |
| 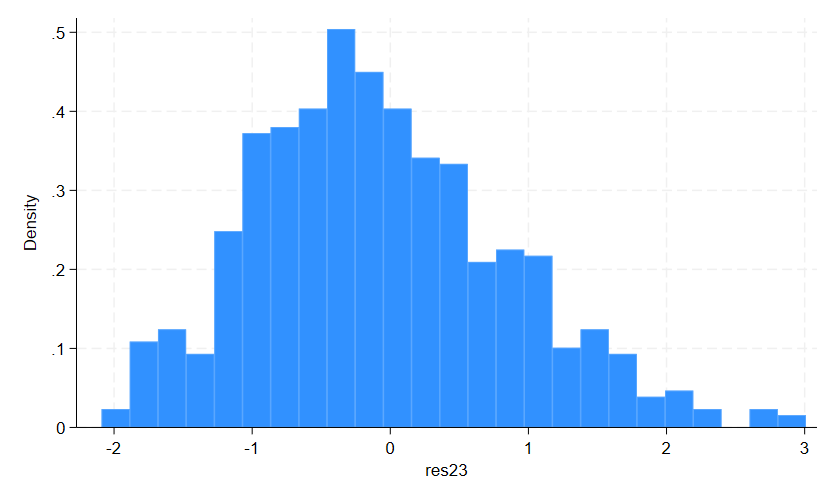 | 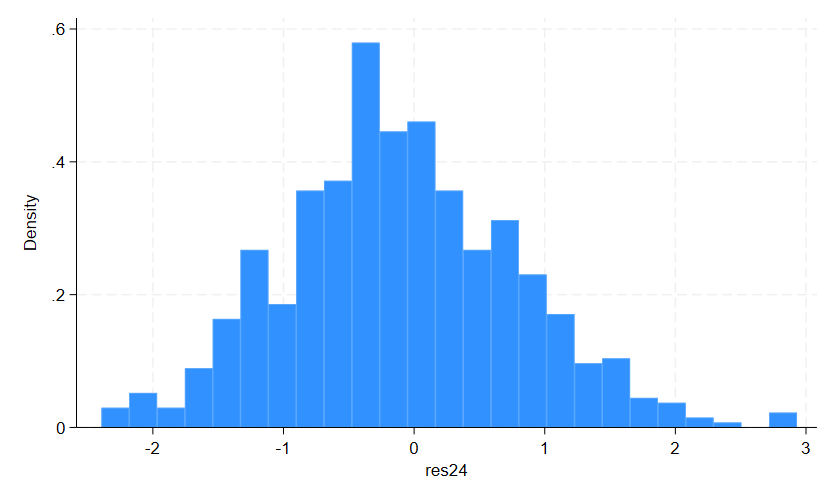 |
